# Common and Distinct Features of Adult Neurogenesis and Regeneration in the Telencephalon of Zebrafish and Mammals

**DOI:** 10.3389/fnins.2020.568930

**Published:** 2020-09-23

**Authors:** Nicolas Diotel, Luisa Lübke, Uwe Strähle, Sepand Rastegar

**Affiliations:** ^1^INSERM, UMR 1188, Diabète athérothrombose Thérapies Réunion Océan Indien (DéTROI), Université de La Réunion, Saint-Denis, France; ^2^Institute of Biological and Chemical Systems-Biological Information Processing (IBCS-BIP), Karlsruhe Institute of Technology (KIT), Karlsruhe, Germany

**Keywords:** BMP, notch, inflammation, brain lesion, neural stem cell, adult neurogenesis, zebrafish, mouse

## Abstract

In contrast to mammals, the adult zebrafish brain shows neurogenic activity in a multitude of niches present in almost all brain subdivisions. Irrespectively, constitutive neurogenesis in the adult zebrafish and mouse telencephalon share many similarities at the cellular and molecular level. However, upon injury during tissue repair, the situation is entirely different. In zebrafish, inflammation caused by traumatic brain injury or by induced neurodegeneration initiates specific and distinct neurogenic programs that, in combination with signaling pathways implicated in constitutive neurogenesis, quickly, and efficiently overcome the loss of neurons. In the mouse brain, injury-induced inflammation promotes gliosis leading to glial scar formation and inhibition of regeneration. A better understanding of the regenerative mechanisms occurring in the zebrafish brain could help to develop new therapies to combat the debilitating consequences of brain injury, stroke, and neurodegeneration. The aim of this review is to compare the properties of neural progenitors and the signaling pathways, which control adult neurogenesis and regeneration in the zebrafish and mammalian telencephalon.

## Introduction

Adult neurogenesis is the process by which neural stem cells (NSCs) divide and provide new neurons which will migrate and differentiate concomitantly in order to establish and/or integrate into existing neural networks of the adult nervous system. During almost one century, the dogmatic view has been held that neurogenesis was restricted to the developmental period and no new neurons were generated during adulthood (Oppenheim, 2019). Step by step, progress in the analysis of the encephalon, cell proliferation and in tracing the genesis of newborn cells, has provided convincing evidence that neurogenesis happens also at post-natal and adult stages (Altman and Das, 1965; Altman, 1969). Post-natal neurogenesis was observed in the cerebellum of mammals, as well as in the subventricular zone (SVZ) of the lateral ventricles and in the dentate gyrus (DG) of the hippocampus (Uzman, 1960; Miale and Sidman, 1961; Altman, 1963, 1969; Altman and Das, 1965). It is nowadays well-established that adult neurogenesis also occurs in the human brain, although its functional significance is still under debate (Boldrini et al., 2018; Sorrells et al., 2018; Oppenheim, 2019). The discovery of adult neurogenesis opened the prospect to repair damage caused by brain injuries (i.e., ischemia and trauma) and neurodegenerative diseases including Parkinson’s and Alzheimer’s diseases in human patients.

In mammals, neuroepithelial cells (NECs) are the first neurogenic cells in the developing nervous system. As the brain ventricles form, these cells differentiate into radial glial cells (RGCs) that have been initially described to support the migration of newborn neurons and to constitute a heterogeneous population (Kriegstein and Gotz, 2003; Pinto and Gotz, 2007). Further studies revealed that RGCs give rise to glial progeny (oligodendrocytes and ependymal cells) but can also behave as NSCs and generate almost all neurons of the brain (Noctor et al., 2002; Malatesta et al., 2003; Spassky et al., 2005; Rowitch and Kriegstein, 2010). In mouse, at the end of the embryonic period some of the RGCs transform into astrocytes (Gaiano et al., 2000; Ventura and Goldman, 2007). The majority of these cells are non-neurogenic, since only RGC-astrocyte-like cells found in discrete regions of the telencephalon, mainly the SVZ of the lateral ventricles and the subgranular zone (SGZ) of the DG of the hippocampus, conserved their neurogenic properties during adulthood (Dennis et al., 2016; Fares et al., 2019).

In contrast to the mammalian brain, RGCs and NECs persist widespread during adulthood in teleost fish and maintain their neurogenic properties (Zupanc, 2001; Zupanc and Clint, 2003; Pellegrini et al., 2007; Kaslin et al., 2009, 2017; Ito et al., 2010; März et al., 2010; Strobl-Mazzulla et al., 2010; Takeuchi and Okubo, 2013; Lindsey et al., 2018). The presence of these numerous NSCs throughout the brain supports an intense neurogenic activity during the entire lifespan (Pellegrini et al., 2005; Zupanc et al., 2005; Lindsey and Tropepe, 2006; März et al., 2010; Edelmann et al., 2013). In addition, while central nervous system (CNS) regeneration is very limited in mammals, adult zebrafish exhibit a huge capability for regenerating the brain (Ayari et al., 2010; Diotel et al., 2010a, 2013; März et al., 2011; Baumgart et al., 2012; Kizil et al., 2012a; Schmidt et al., 2013). These features highlight the prospect that zebrafish could represent an alternative model for a better understanding of constitutive and regenerative neurogenesis in the vertebrate brain.

The aim of this review is to describe the neurogenic and regenerative features of the adult zebrafish brain and to compare them with the mouse. We first describe the ontogeny, the anatomy and the proliferative areas within the telencephalon of zebrafish, considering that this part of the encephalon shares numerous homologies with mammals. The different types of progenitors within the telencephalon, their type of division and their lineage will be emphasized. In a second part, we compare the molecular mechanisms orchestrating constitutive neurogenesis and regeneration in the telencephalon with a focus on Notch, BMP, and inflammatory signaling pathways.

## Ontogenesis of the Zebrafish Central Nervous System

**FIGURE 1:**
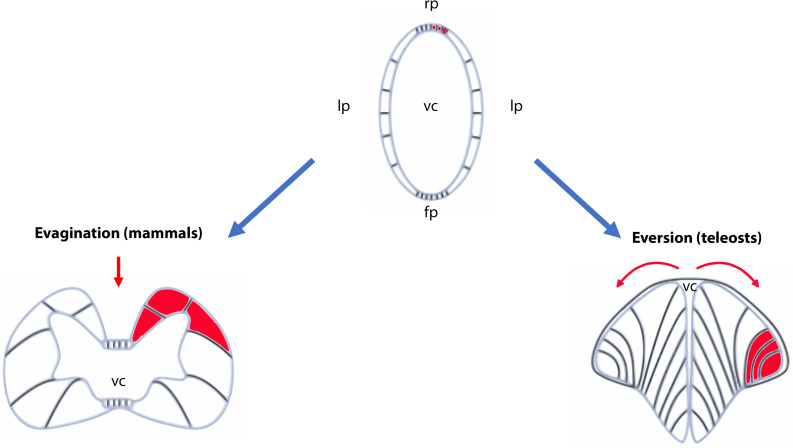
Schematic representation of evagination and eversion in vertebrates. The top panel illustrates the telencephalic part of the neural tubes that will evolve differently during development between ray-finned fish (actinopterygians) and other vertebrates. In the latter, the neural tube will follow an evagination process of the telencephalic vesicles (left panel) producing paired telencephalic hemispheres with two internal ventricles (the lateral ventricles in mammals). In contrast, in ray-finned fish (right panel), the neural tube in its dorsal region (pallium) grows and curves laterally. It folds toward the ventral region (subpallium) producing two massive hemispheres flanking a single ventricular cavity. Such movements will stretch the dorsal roof-plate region of the neural tube and will form the tela choroidea. The red parts show the different positioning of the territories following the eversion or evagination processes. The red arrows highlight the movements occurring in the neural tubes. Fp, floor plate; lp, lateral plate; rp, roof plate; vc, ventricular cavity.

The development of the CNS starts at around 6 h post fertilization (hpf) corresponding to the beginning of gastrulation (Woo and Fraser, 1995). The main brain structures are initially heralded by region-specific expression of neural genes (Bally-Cuif and Hammerschmidt, 2003; Mueller and Wulliman, 2005). At 24 hpf, the forebrain, the midbrain and the hindbrain have formed morphologically distinct structures. Later during development, these embryonic structures will differentiate and generate the main structures of the adult brain, including the telencephalon, the diencephalon and the rhombencephalon. It is also important to note, that in contrast to mammals, the telencephalon develops by eversion and not by evagination (Mueller and Wulliman, 2005; Folgueira et al., 2012). This specific type of development of the pallium makes the identification of homologous regions shared with the mammalian telencephalon more difficult, notably concerning the hippocampal neurogenic region. In addition, instead of having two telencephalic ventricles (the two lateral ventricles in mammals), there is only one medial ventricle in teleost fish (Figure 1).

## Anatomy of the Zebrafish Telencephalon Shows Resemblance to the Mouse Telencephalon Despite Different Modes of Ventricle Formation

**FIGURE 2:**
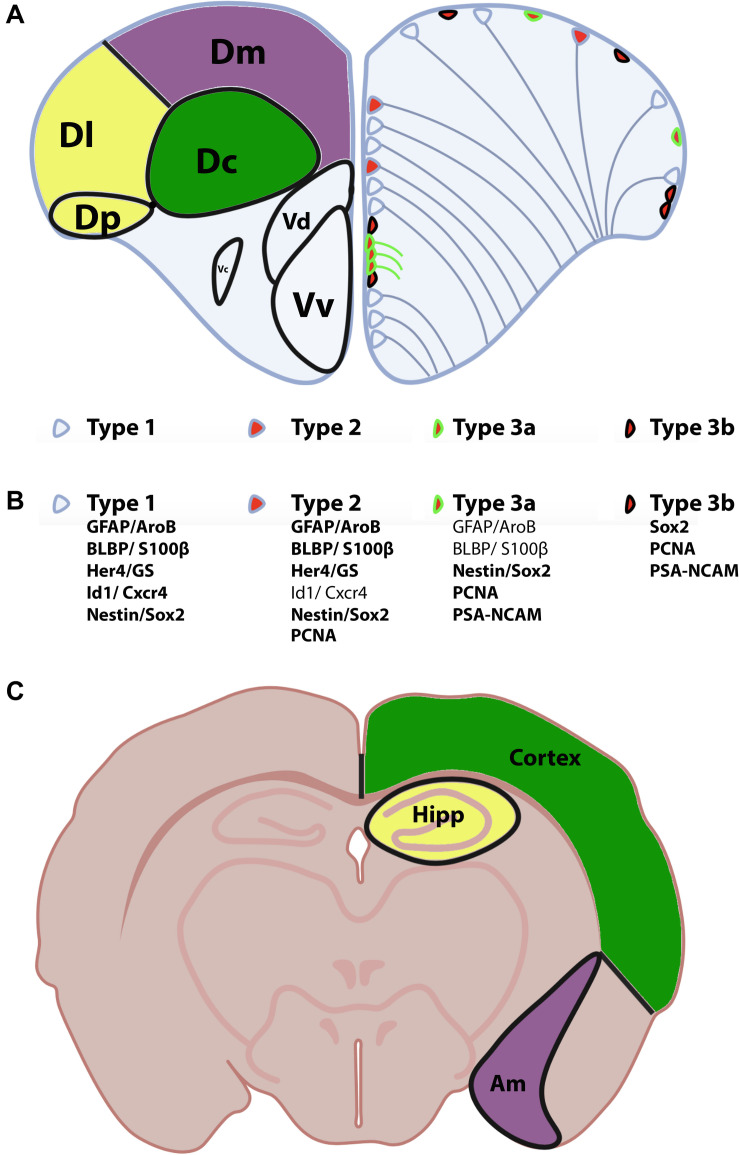
Anatomy of the zebrafish telencephalon and homologies with the mammal brain. **(A)** Zebrafish telencephalon indicating the different brain regions and/or nuclei of the pallium and subpallium in the left hemisphere according to Wullimann et al. (1996). The Dm (purple), Dl/Dp (yellow), and Dc (green) have been proposed to be the homologs of the amygdala, hippocampus and the neocortex in rodents (see corresponding colors in the bottom scheme C). The right zebrafish telencephalic hemisphere illustrates the distribution of neural stem/progenitor cells with type 1 (quiescent RGCs), type 2 (proliferative RGCs) and type 3 cells (neuroblasts)**. (B)** Molecular features/markers of type 1, 2, and 3 cells are indicated below the scheme. Bold writing corresponds to strong expression of the proteins. **(C)** Mouse transversal section through the telencephalon and diencephalon indicating the corresponding homologies with the zebrafish telencephalon. Am, amygdala; Dc, central zone of the dorsal telencephalon; Dl, lateral zone of the dorsal telencephalon; Dm, dorsomedial zone of the dorsal telencephalon; Dp, posterior zone of the dorsal telencephalon; Hipp, hippocampus; Vv, ventral nucleus of the ventral telencephalon; Vd, dorsal nucleus of the ventral telencephalon.

In zebrafish, the telencephalon is composed of the olfactory bulbs, the subpallium (ventral telencephalon) and the pallium (dorsal telencephalon). The ventral telencephalon is subdivided into two brain nuclei: the ventral nucleus of the ventral telencephalon (Vv) and the dorsal nucleus of the ventral telencephalon (Vd) (Wullimann et al., 1996; Figure 2A). The dorsal telencephalon is more complex and is composed of different brain nuclei and/or regions including the central zone (Dc), the dorsomedial zone (Dm), the lateral zone (Dl) and the posterior zone (Dp) of the dorsal telencephalon (Wullimann et al., 1996; Figure 2A). These different brain nuclei/regions occupy distinct rostro-caudal positions. Interestingly, the telencephalon contains several neurogenic niches (estimated to 16 according to Byrd and Brunjes, 2001; Adolf et al., 2006; Grandel et al., 2006; Kishimoto et al., 2011, 2013). Among these, the Vv of the subpallium is considered to be homologous of the SVZ of the lateral ventricle of mammals, and the Dl and/or Dp of the pallium to be the equivalent of the SGZ of the DG (see below for description of these neurogenic niches) (Broglio et al., 2005; März et al., 2010; Grandel and Brand, 2013; Ganz et al., 2014). However, these homologies between fish and rodents would benefit from further investigation in order to ascertain such evolutionary comparisons.

The work from Ganz et al. (2014) aimed at identifying the expression pattern of conserved genes in the pallium of adult zebrafish in order to define pallial subdivisions and to determine their homologs in tetrapods (Ganz et al., 2014). They suggested that the Dm corresponds to the pallial amygdala in mammals, the Dc is the homolog of the cortex and the Dl (ventral and dorsal parts) could be the homolog of the hippocampus. In their work, Ganz et al. (2014) did not find an equivalent of the Dp in tetrapods.

In addition, by performing *in situ* hybridization for 1202 transcription regulators (TRs) in the telencephalon of adult zebrafish, we previously identified particular regions displaying specific clusters of TR gene expression revealing similarities with the mammalian brain (Diotel et al., 2015b). Among the 1202 TRs analyzed, 562 exhibited 13 distinct patterns and some of them were restricted to the neurogenic niches localized along the telencephalic ventricle. In the same line, neurotransmitters, their synthesizing enzymes, and specific markers for GABAergic neurons in the telencephalon or serotoninergic innervation of the telencephalon (or serotoninergic neurons in other brain regions than the telencephalon) are expressed in a very similar way to what is observed in rodents, suggesting once again homologies with the mammalian telencephalic regions during development and adulthood (Kaslin and Panula, 2001; Mueller et al., 2004; Mueller and Wullimann, 2009; Wullimann, 2009; Parker et al., 2013). Thus, the zebrafish subpallial regions have been suggested to be the homologs of the medial and lateral ganglionic eminences in mouse but further investigation is needed to confirm this hypothesis (Mueller and Wullimann, 2009).

In summary, the telencephalon of adult zebrafish shares similarities in its subdomains with those of mouse regarding the expression of transcription regulators, the types and the distribution of excitatory and inhibitory neurons, as revealed by the expression of their respective neurotransmitter synthesizing enzymes. The homologies between the different telencephalic regions according to the expression of TRs have been highlighted in the Figures 2A,C. However, the different models based on gene expression require a unifying theory, which may at the end only be possible to derive by including functional aspects of the various regions.

## Proliferative Areas in the Adult Telencephalon

**FIGURE 3:**
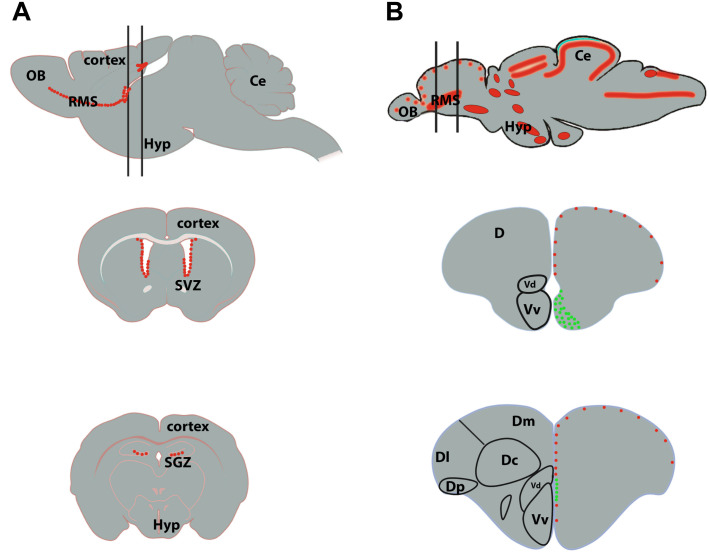
Neurogenic niches in the brain of mammals and zebrafish. **(A)** Sagittal section of a rodent brain illustrating the main proliferative area shown by red dots (top scheme). The two black lines correspond to coronal sections through the subventricular zone (SVZ) of the lateral ventricles (middle scheme) and the subgranular zone (SGZ) of the dentate gyrus of the hippocampus (bottom scheme). The red dots correspond to proliferative cells. **(B)** Sagittal brain section of a zebrafish brain illustrating the main proliferative areas shown by red dots (top scheme). The two black lines correspond to coronal sections through the anterior part of the telencephalon, where the ventricular zone of the Vv-Vd is suggested to be the equivalent of the SVZ in mammals (middle scheme), and through the medial part of the telencephalon, where the Dl/Dp is suggested to be the homolog of the hippocampus in mammals (bottom scheme). In zebrafish, the red dots correspond to slow cycling progenitors (mainly RGCs, type 2) and the green ones to fast cycling progenitors (mainly neuroblasts, type 3). Ce, cerebellum; D, telencephalic dorsal area; OB, olfactory bulbs; Hyp, hypothalamus.

The brain of adult teleost fish displays many proliferative areas throughout its rostrocaudal extend (Zupanc et al., 2005; Lindsey and Tropepe, 2006; Pellegrini et al., 2007; Figure 3). These proliferative sites correspond essentially to neurogenic niches and are mainly located along the ventricular layers of the telencephalon, diencephalon (thalamus and hypothalamus) and rhombencephalon. For its ease of experimental access, the zebrafish telencephalon is one of the most studied parts of the brain regarding neurogenesis (Grandel et al., 2006; Lam et al., 2009; März et al., 2010).

We will next describe the localization of the different neurogenic niches in zebrafish and mouse (Figure 3). Adolf et al. (2006) demonstrated the existence of slow and fast cycling progenitors in the telencephalon of adult zebrafish (Figure 3). In the ventral telencephalon, fast cycling progenitors were observed in the ventricular zone of the Vv in a wide band composed of densely packed proliferating cells (Adolf et al., 2006; Ganz and Brand, 2016; Figure 3B, middle scheme). They display interkinetic nuclear migration and do not or only barely express typical glial markers (Ganz et al., 2010; März et al., 2010). These fast cycling progenitors were shown to correspond to neuroblasts migrating tangentially toward the olfactory bulbs through a rostral migratory stream-like (Kishimoto et al., 2011, 2013). More caudally, these fast cycling progenitors were observed in a thinner band localized more dorsally (Grandel et al., 2006; März et al., 2010; Lindsey et al., 2012; Ganz and Brand, 2016; Figure 3B, bottom scheme). Another small region in the posterior zone (Dp) of the pallium is also composed of fast cycling progenitors. In contrast, the dorsal subpallium (Vd), as well as the rest of the ventricular layer of the dorsal telencephalon are mainly composed of slow cycling progenitors (Adolf et al., 2006; März et al., 2010; Figure 3).

In mouse, only two main regions exhibit a significant level of proliferation in the telencephalon: the SVZ of the lateral ventricles and the SGZ of the DG in the hippocampus. Numerous cycling cells originate from the SVZ and migrate tangentially toward the olfactory bulbs forming the rostral migratory stream (RMS) (Figure 3A; Doetsch et al., 1997). These migrating cells in proliferation correspond to neuroblasts in zebrafish. Thus, the SVZ and RMS have their equivalents in the zebrafish telencephalon with the RMS-like structure composed of neuroblasts originating from the cluster of fast-cycling densely packed neural progenitors localized in the zebrafish subpallium (Vv; Figure 3B, middle scheme, in green) (Adolf et al., 2006; März et al., 2010; Kishimoto et al., 2011). In the mouse hippocampus, the SGZ also harbors proliferative cells corresponding to NSCs, intermediate progenitors and neuroblasts (Kozareva et al., 2019). These cells will provide new neurons to the hippocampal formation. The hippocampal NSCs display radial glial like characteristics and share features with zebrafish quiescent and proliferative RGCs from the pallium (see below).

In conclusion, strong homologies exist between the neurogenic niches in zebrafish and the mouse telencephalon: (1) studies of functional and molecular markers identified the zebrafish Dp/Dl as the homolog of the hippocampus in mouse; (2) the Vv, composed of densely packed fast cycling progenitors, has been proposed to be the equivalent of the SVZ/RMS in mouse.

## The Different Types of Progenitors in the Telencephalon and Their Lineage

**FIGURE 4:**
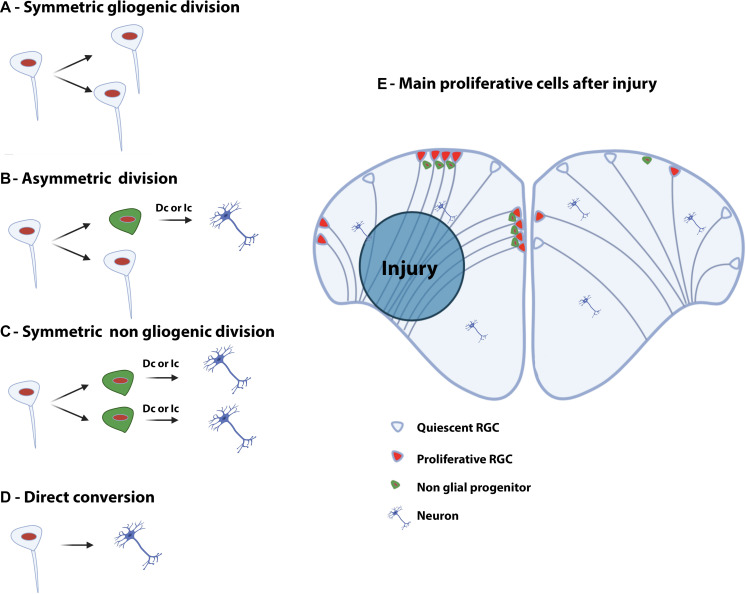
Type of divisions of neural stem/progenitor cells and injury-induced proliferation in the telencephalon of adult zebrafish. **Left panel:** Symmetric gliogenic division **(A)** in which one dividing RGC provides two new RGCs. A RGC can also divide asymmetrically **(B)**, self-renew and generate a non-glial progenitor (neuroblast). Another possible type of division is the symmetric non-gliogenic division **(C)** in which one RGC provides two non-glial progenitors. Finally, direct conversion of a RGC into a neuron can also occur **(D)**. The non-glial progenitors can give rise to new neurons through direct conversion (Dc) or indirect conversion (Ic). **Right panel:** Injury-induced proliferation following traumatic injury in the brain of adult zebrafish **(E)**. In the intact hemisphere (right part), most RGCs are quiescent (type 1) and some are proliferating (type 2). There are also a number of non-glial progenitors in the pallium. Five days after stab wound injury (left part), RGCs actively divide and generate numerous non-glial progenitors (type 3) that will provide new neurons to replace the damaged and dying ones.

The work from März et al. (2010) suggested the existence of three main types of neurogenic proliferative cells in the telencephalon of adult zebrafish: type 1, type 2, and type 3 (März et al., 2010; Figures 2A,B). Type 1 and type 2 are NSCs and correspond to quiescent (qNSCs) and activated proliferative RGCs (aNSCs), respectively, while type 3 (a and b) corresponds to further committed progenitors supposed to be neuroblasts. In zebrafish, type 1 cells are negative for the proliferating cell nuclear antigen (PCNA) and express the whole set of RGC markers (i.e., S100β, GFAP, BLBP, AroB, and Vimentin), as well as the progenitor markers Sox2 and Nestin (Figure 2B). It is nevertheless important to mention that Nestin is not strongly expressed by RGCs, given that *in situ* hybridization experiments and transgenic BAC lines do not recapitulate the expression pattern shown by a transgenic construct containing a short promoter sequence (Mahler and Driever, 2007; Kaslin et al., 2009; Lam et al., 2009). Additionally, *sox2* expression is also detected in neurons and oligodendrocyte progenitor cells and is therefore not specific to a certain cell population. In contrast, type 2 cells display the same set of RGC and progenitor markers but are positive for PCNA (Figure 2B). They are consequently proliferative NSCs. Two different subsets of type 3 progenitors have been identified: type 3a and type 3b. Type 3a do not or only weakly express RGC markers, but are positive for the progenitor marker Sox2 and the Nestin short transgene (März et al., 2010). Additionally, they are in a proliferative state (PCNA-positive) and express PSA-NCAM, a marker of early neuronal differentiation. In contrast, type 3b do not express any of the RGC markers, and are only positive for the progenitor marker Sox2. However, they are also in a proliferative state (PCNA-positive) and express PSA-NCAM (März et al., 2010; Figure 2B). Using GFP-encoding retrovirus and lentivirus tracing and genetic lineage tracing it was shown that RGCs can provide different types of neurons and confirm a lineage between type 1, 2, and 3 (Rothenaigner et al., 2011; Lange et al., 2020; Than-Trong et al., 2020). This and more recent works revealed that RGCs can go through symmetric gliogenic division, asymmetric division producing RGCs and neuroblasts (type 3), and that RGCs can undergo direct conversion into neurons (Rothenaigner et al., 2011; Barbosa et al., 2015; Figure 4). These studies demonstrated that RGCs exhibit features of true NSCs being able to self- renew, in order to maintain a pool of stem cells. In contrast, type 3 cells mainly perform symmetric neurogenic divisions (Rothenaigner et al., 2011).

In the adult mouse SVZ, NSCs (B cells) display astrocytic features and provide transit amplifying cells (C cells). These cells can generate neuroblasts (A cells). The quiescent B cells express GFAP and Nestin but are negative for neuroblast markers such as doublecortin (DCX). The transit amplifying cells (C cells) weakly express GFAP, and are strongly Nestin- and PCNA-positive but still negative for neuroblast markers (e.g., DCX). In sharp contrast, the A cells, corresponding to neuroblasts, do not express GFAP and Nestin anymore, but are strongly positive for DCX and PCNA (Cavaliere et al., 2016).

In the adult SGZ of the hippocampus, the quiescent (type 1) and proliferative (type 2) NSCs display radial glial-like morphology. Type 2 cells give rise to type 3 cells corresponding to neuroblasts that will provide new neurons. Type 1 cells express GFAP, Nestin, BLBP and Sox2 while type 2 cells show a decreased expression of GFAP but are positive for PCNA. Type 3 cells express further committed markers such as DCX and PSA-NCAM in addition to being PCNA-positive (Knoth et al., 2010; Kozareva et al., 2019).

Consequently, morphological and lineage features of progenitors from the dorsal pallium in zebrafish are very similar to those from the SGZ in the hippocampus of mouse in which type 1 progenitors (quiescent radial glial-like cells) can enter a proliferative state (type 2) and give rise to neuroblasts (type 3), providing new neurons (Knoth et al., 2010; Kozareva et al., 2019).

The SVZ-RMS in mouse also shares characteristics with the zebrafish-equivalent of the Vv-RMS. Differences exist, however, in the way of migration: the neural progenitors migrate along blood vessels in fish and not along scaffolds formed by glial tubes, as observed in mouse (Kishimoto et al., 2011).

## The Cellular and Molecular Morphology of NSCs and the Absence of Astrocytes in Fish

An interesting point to be highlighted is the nature of adult NSCs in the brain of zebrafish and mouse; in zebrafish, the *bona fide* NSCs are RGCs, in mouse, adult NSCs are either radial-glial like cells (type 1 cells in the SGZ) or cells exhibiting astrocytic features (B cells in the SVZ) (Morrens et al., 2012; Dimou and Gotz, 2014). RGCs are derived from NECs. In both prenatal mammals and in fish from embryonic stages to adulthood, RGCs, and NECs have a similar morphology. They display a small triangular or ovoid soma localized close to the ventricle, and extend two cytoplasmic processes: one long process crossing the brain parenchyme to reach the pial surface, and one short process toward the ventricular surface. These NSCs also serve as a scaffold for the migration of newborn neurons (Noctor et al., 2002; Weissman et al., 2003). At the end of the embryonic development in mouse, RGCs disappear and transform mainly into astrocytes. In contrast, the brains of adult teleost do not contain astrocytes but maintain RGCs and NECs during adulthood sustaining the strong neurogenic activity of fish (Noctor et al., 2002; Merkle et al., 2004; Pinto and Gotz, 2007; Kaslin et al., 2009; Than-Trong and Bally-Cuif, 2015). The adult telencephalic neurogenic activity is consequently mainly due to the maintenance of (1) RGCs in the dorsal telencephalon and (2) NECs in the ventral telencephalon.

Both, embryonic mouse RGCs and adult zebrafish RGCs, express evolutionary conserved markers, such as the intermediate filaments GFAP (glial fibrillary acidic protein) and vimentin, the brain lipid binding protein (blbp), the calcium binding protein (S100β), the estrogen-synthesizing enzyme Aromatase, and progenitor markers like Nestin and Sox2 (Pellegrini et al., 2007; März et al., 2010; Lindsey et al., 2012; Than-Trong and Bally-Cuif, 2015; Diotel et al., 2016; Figure 2B). In zebrafish, RGCs express additional markers such as the *inhibitor of DNA binding 1* (*id1*) and the chemokine receptor *cxcr4*, both of them being mainly expressed in type 1 cells and only in a few type 2 cells (Diotel et al., 2010b; Rodriguez Viales et al., 2015). Alike, *Id1* and *Cxcr4* have been shown to be expressed in RGCs and/or adult NSCs in mouse (Tran et al., 2007; Nam and Benezra, 2009; Mithal et al., 2013; Ho et al., 2017). Several *her* genes, such as *her4*, the downstream target genes of Notch signaling, are expressed in the telencephalic neurogenic niches, especially in RGCs (Chapouton et al., 2011; Kroehne et al., 2011; Diotel et al., 2015b). Similarly, the orthologs of the zebrafish *her*, the *Hes* genes are expressed in RGCs in mouse and are involved in NSC fate choice (see Table 1). Most RGC markers are also synthesized by adult NSCs of the SVZ and SGZ in mouse, underscoring that zebrafish and mouse adult NSCs are very similar. In their recent review, Labusch et al. (2020) also detailed that RGCs from the pallium display strong cellular and molecular similarities with the adult NSCs of rodents.

**TABLE 1:** Summary of key molecules involved in adult neurogenesis and regeneration.

Pathways	Molecule	Role in zebrafish adult neurogenesis	References	Role in mouse/mammalian adult neurogenesis	References
**Constitutive neurogenesis**					
Notch	Notch1	Notch1b ortholog, expressed in activated progenitors, function: maintains progenitor state	Alunni et al., 2013	Expressed in activated NSCs of the SVZ, function: proliferation	Basak et al., 2012
	Notch3	Expressed in quiescent and activated RGCs, function: controls proliferation in interaction with Hey1 and quiescence in interaction with Her4	Alunni et al., 2013; Than-Trong et al., 2018	Expressed in quiescent NSCs in the SEZ, function: maintenance of quiescent NSCs	Kawai et al., 2017
	Hes1	Her6 ortholog, expressed in qRGCs of the VZ	Chapouton et al., 2011	Function: repression of proneural genes, maintenance of NSCs	Boareto et al., 2017
	Hey1	Expressed in all progenitor cells in adult pallium, function: interaction with Notch3 to drive proliferation	Than-Trong et al., 2018	Not analyzed in mouse	–
	Her4	Expressed in RGCs, function: interaction with Notch3 to drive quiescence	Alunni et al., 2013	Hes5 ortholog, expressed in RGCs, function: represses NSC differentiation	Kageyama et al., 2007b
	Ascl1	Ascl1a ortholog, expressed in activated NSCs	Than-Trong et al., 2018	Proneural gene, function: constant expression leads to differentiation of NPCs to neuroblasts, oscilating expression leads to maintenance of proliferative state	Imayoshi et al., 2013
BMP	BMP	Function: control of *id1* expression in RGCs through BRE	Zhang et al., 2020	Signaling active in NSCs, function: balances quiescence and proliferation	Mira et al., 2010
	Noggin	No data reported in adult neurogenesis	–	Expressed by ependymal cells close to the SVZ, function: antagonist of BMP	Lim et al., 2000
	BMP specific Smads	Mediators of BMP pathway, function: regulation of *id1* expression in RGCs	Zhang et al., 2020	Active in stem/progenitor cells, function: mediators of the canonical BMP pathway	Mira et al., 2010
	Smad4	Co-Smad, function: together with BMP specific Smads regulation of *id1* expression	Zhang et al., 2020	Function: deletion of Smad4 leads to blockage of the BMP pathway and enhanced neurogenesis	Mira et al., 2010
	Id1	Expressed in qNSCs, function: promotes NSC quiescence	Rodriguez Viales et al., 2015	Expressed in B1- type neural stem cells, function: stem cell self- renewal	Nam and Benezra, 2009
**Reactive neurogenesis**					
BMP	BMP	Delayed activation of BMP/Id1 after injury to promote quiescence and avoid depletion of stem cell pool	Rodriguez Viales et al., 2015	Upregulation of BMP and downstream effector Id3 in SVZ upon injury, function: interaction of Id3 and E47 leads to differentiation of NSPCs into astrocytes	Bohrer et al., 2015
Notch	Notch1	Upregulated in NSPCs, function: injury-induced proliferation	Kishimoto et al., 2012	Induced in response to injury in SVZ, function: increases astrogenic response of NSCs	Tatsumi et al., 2010; Benner et al., 2013
Inflammation	Gata3	Injury-induced increase in expression in NSCs, function: promotes proliferation and differentiation into neurons	Kizil et al., 2012b	Gata3 is unable to induce neurogenesis in 3D cultures of human astrocytes	Celikkaya et al., 2019
	LtC4	Function: together with receptor CysLt1, activation of *gata3* in injured telencephalon	Kyritsis et al., 2012	Not analyzed in mouse	–
Amyloid beta induced Alzheimer’s Model (inflammation)	IL4	Function: interaction with STAT6 (downstream effector) to promote RGC proliferation and development into neurons	Bhattarai et al., 2016	Function: in mouse no neurogenesis observed in astroglia upon induction of type 1 IL4 signaling Human astroglia in 3D culture form neurons upon induction of type 2 IL4 signaling	Mashkaryan et al., 2020 Papadimitriou et al., 2018
	BDNF	Function: required for proliferation and neurogenesis of NSCs	Bhattarai et al., 2020	Function: In injured rat brain BDNF promotes recruitment, neuronal differentiation and survival of progenitor cells	Henry et al., 2007

An interesting evolutionary aspect to stress is that astrocytes derived from RGCs in mouse express markers like GFAP, vimentin, glutamin synthase and S100β, which are also expressed by zebrafish RGCs (März et al., 2010; Kroehne et al., 2011; Dimou and Gotz, 2014; Wang et al., 2019). The fact that the brain, in particular the telencephalon, of zebrafish is devoid of astrocytes, raises the question of which cells adopted their astrocytic functions [i.e., in blood brain barrier (BBB) establishment, neurotransmitter uptake, ionic regulation, and neurosteroidogenesis] (Jurisch-Yaksi et al., 2020). More and more data in fish point to the general idea that zebrafish RGCs fulfill the functions of astrocytes. For instance, in zebrafish, adult RGC endfeet wrap the blood vessels (Diotel et al., 2010b, 2018), arguing for a role of RGCs in BBB physiology, as the mechanism is comparable to astrocytic endfeet wrapping around endothelial cells in mammals. RGCs also seem to express a whole set of steroidogenic enzymes, in a way similar to what is known in mammals (Diotel et al., 2011, 2018; Pellegrini et al., 2016; Weger et al., 2018). In addition, the main water channel of the brain, aquaporin-4 (*AQP4*), is expressed by astrocytes in mammals and by RGCs in zebrafish (King et al., 2004; Grupp et al., 2010), but their cellular distribution differs. To better understand other evolutionary conserved aspects between zebrafish and mammals, such as ionic regulation and neurotransmitter uptake, further studies are necessary. It is also important to note that aromatase, expressed in RGCs during development in mammals and during both development and adulthood in zebrafish, is not anymore expressed in astrocytes, except upon brain injury in reactive astrocytes (Garcia-Segura et al., 1999; Kah et al., 2009; Diotel et al., 2010a). In contrast, after brain injury in fish, aromatase expression is decreased in RGCs and in the injured telencephalon, while *de novo* expression seems to occur in cells resembling neurons (Diotel et al., 2013).

Thus, the different types of neural progenitors found in the main telencephalic neurogenic niches share markers conserved between zebrafish and mice, during both development and adulthood, supporting further the importance of zebrafish as an ideal animal model in understanding vertebrate NSC properties. Thus, zebrafish RGCs exhibit cellular and molecular similarities with adult NSCs localized in the mammalian SVZ and SGZ. However, some differences exist regarding, for instance, the morphology of NSCs during adulthood and their mode of division. Indeed, zebrafish NSCs perform symmetric divisions and asymmetric divisions (Rothenaigner et al., 2011; Than-Trong and Bally-Cuif, 2015; Than-Trong et al., 2020). Another interesting feature is the direct conversion of RGCs into neurons which seems to represent an important method to generate neurons in the adult zebrafish brain (Barbosa et al., 2015). This differs from the behavior of NSCs in adult mouse: Murine NSCs are characterized by asymmetric divisions and the genesis of intermediate progenitors (Suh et al., 2007; Costa et al., 2011; Than-Trong et al., 2018).

## Heterogeneity in the RGC and Progenitor Population

RGCs do not appear to constitute a homogeneous population. Although, zebrafish RGCs express a wide set of markers including Nestin, GFAP, BLBP, Cyp19a1b (Aromatase B), S100β, vimentin, and glutamin synthase (Pellegrini et al., 2007; Lam et al., 2009; Diotel et al., 2010a, 2016; März et al., 2010; Kroehne et al., 2011), these markers are not 100% co-expressed in all RGCs (Figure 2). For instance, despite the fact that a wide proportion of RGCs co-express Aromatase B and BLBP, some of them either express Aromatase B or BLBP throughout the brain, while BLBP expression is associated with a higher proliferative state of RGCs (Diotel et al., 2016). Additionally, the chemokine receptor *cxcr4* appears to be co-expressed with Aromatase B and BLBP, but some RGCs express these markers differentially, which suggests regionalization, as in the periventricular pretectal nucleus and along the lateral and posterior hypothalamic recess (Diotel et al., 2010b, 2016). Indeed, in these regions, some parts are only Aromatase B-positive, or BLBP-positive or co-express both markers. The same was observed for Cxcr4. These data suggest the existence of heterogeneity between RGCs throughout the brain but also within the telencephalon (Figure 2). In addition, a recent review documents the heterogeneity within the neural progenitor population in zebrafish with some progenitors expressing genes involved in astroglial functions and others expressing typical ependymal markers (Jurisch-Yaksi et al., 2020). Also, among pallial RGCs, some seem to be deeply quiescent and others appear to constitute a self-renewing reservoir (Than-Trong et al., 2020). This also supports the idea of heterogeneity within the RGC population.

In a different way, these differences in gene expression are underscored by an unbiased analysis of transcription regulators (TRs) expression in the telencephalon of adult zebrafish (Diotel et al., 2015b). Our comprehensive, genome-wide expression mapping of TRs in the telencephalon of adult zebrafish showed that certain TRs display a restricted expression pattern in neurogenic niches localized along the telencephalic ventricle (Diotel et al., 2015b). For instance, the *SRY-box containing gene 4a* (*sox4a*) is detected in the ventricular and periventricular layer of the RMS, the *POU class 3 homeobox 3b* (*pou3f3b*) gene is strongly expressed in the RMS and also weakly in the ventricular zone, while the *Forkhead box J1a* (*foxj1a*) gene is specifically expressed in the RMS. In addition, TRs such as *doublesex and mab-3 related transcription factor like family a2* (*dmrta2*) are expressed specifically in the ventricular zone but not in the RMS. These results suggest the existence of specific programs controlling NSC activity with distinct neurogenic outcomes. The hierarchal clustering of genes expressed in specific regions of the VZ clearly shows that the VZ neurogenic region is not homogeneous between the medial part and the lateral part of the VZ. Interestingly, such differences in expression can as well be found in a comparative approach in mammals. For instance, in the rodent brain, *FoxJ1* expressing cells produce neuroblasts and are implied in postnatal neurogenesis (Jacquet et al., 2011; Devaraju et al., 2013; Muthusamy et al., 2014). Additionally, Pou3f3 modulates neurogenesis in the ventricular zone in mouse (Dominguez et al., 2013). Consequently, TR gene expression in the telencephalic neurogenic niches of adult zebrafish could share similarities with their homologous regions in the telencephalon of rodents, as described above for the TRs Foxj1 and Pou3f3. Furthermore, it appears that the ventricular zone of the telencephalon exhibits different subdomains considering TR gene expression. This argues in favor of distinct neurogenic and/or gliogenic regions within the pallial ventricular zone (Diotel et al., 2015b). Studies performed in rodents also suggest the existence of different neurogenic domains within the adult SVZ correlated with the expression of different subsets of TRs and linked to different neurogenic properties (Merkle et al., 2007, 2014; Rushing and Ihrie, 2016).

In summary, these observations argue in favor of different subpopulations of RGCs along the telencephalic ventricular zone. Until now, the significance of such differences is not well understood but could be explained by differential neurogenic versus gliogenic properties: proliferation rate, quiescence, varying regenerative properties, as well as the genesis of diverse types of neurons. Such heterogeneity in RGCs was also observed during development in mammals (Pinto and Gotz, 2007). Furthermore, recent data demonstrated that adult NSCs from the SVZ and SGZ in rodents do not constitute a homogeneous population, based on morphological and functional criteria (Gebara et al., 2016; Obernier and Alvarez-Buylla, 2019). Consequently, NSCs in the brain of both adult mammals and fish retain functional similarities and display heterogeneity in gene expression. However, the functional significance of this heterogeneity is not yet understood.

## RGCs Sustain the Strong Regenerative Capacity of the Zebrafish Telencephalon

In addition to displaying a strong constitutive neurogenic activity, zebrafish is also an interesting model for studying regenerative neurogenesis and the behavior of NSCs upon injury due to its high regenerative capability (Alunni and Bally-Cuif, 2016). Directly after mechanical injury of the telencephalon, cell death is observed as early as 4 hpl (hours post lesion) within the damaged hemisphere and starts to reach normal levels from 1 to 3 dpl (days post lesion), according to the type of lesion inflicted (Kroehne et al., 2011; Kyritsis et al., 2012). It also leads to a significant brain oedema at 1 dpl (Kroehne et al., 2011). Shortly after brain injury, microglia cells and peripheral immune cells, close to the damaged regions, are activated and recruited. By using a transgenic fish line [Tg(ApoE-GFP)] and performing immunohistochemistry (L-plastin and 4C4 antibodies), these cells were shown to be recruited starting from 6 hpl on, peaking between 1 and 3 dpl before returning to basal levels at around 7 dpl (Kroehne et al., 2011; März et al., 2011; Baumgart et al., 2012; Kyritsis et al., 2012). Oligodendrocytes and oligodendrocyte precursor cells (OPCs) also accumulate in the injured region from 1 to 14 dpl (März et al., 2011). Interestingly, Olig2-positive cells do not increase their proliferation rate following brain injury, which is in contrast to the mammalian situation (März et al., 2011; Baumgart et al., 2012).

In addition, ventricular proliferation occurs after injury to the telencephalon, but appears to be primarily restricted to the damaged hemisphere (Kroehne et al., 2011; März et al., 2011). From 1 to 3 dpl, most proliferative cells are found in the brain parenchyme. Still, from 48 hpl, proliferation starts to be upregulated along the ventricular layer the neurogenic niche (März et al., 2011; Kishimoto et al., 2012; Diotel et al., 2013). Comparing control and injured hemispheres, periventricular proliferation peaks between 6 and 8 dpl and slowly returns to basal levels around 15 dpl to 35 dpl (März et al., 2011; Kishimoto et al., 2012; Diotel et al., 2013). These reactive proliferating cells, localized along the telencephalic ventricle, are reported to be RGCs, expressing their typical markers such as S100β, BLBP, GFAP, and vimentin (Kroehne et al., 2011; März et al., 2011; Baumgart et al., 2012; Kizil et al., 2012b; Diotel et al., 2013). They give rise to newborn neurons (HuC/D-positive) persisting more than 3 months and expressing mature neuronal and dendritic markers like microtubule-associated protein 2a/b, the synaptic vesicle marker 2 and metabotropic glutamate receptor 2 (MAP2a/b, SV2, and mGlu2, respectively), proving their functional maturation more than 2 to 3 months after brain injury (Kroehne et al., 2011; Baumgart et al., 2012). As for constitutive neurogenesis, lineage-tracing experiments and immunohistochemistry studies have established that newly generated neurons are also generated by RGCs and by type 3 progenitors through different mechanisms (direct conversion of RGCs into neurons, asymmetric division and symmetric non-gliogenic division, Figures 4B–D; Kroehne et al., 2011; Baumgart et al., 2012; Barbosa et al., 2015). In addition, a shift in the mode of division of NSCs has been suggested between intact and injured conditions (symmetric gliogenic division versus symmetric non-gliogenic divisions, Figures 4A,C; Barbosa et al., 2015). Interestingly, after brain injury NSCs mainly performed symmetric divisions giving rise to new neurons, which little by little depletes the NSC pool. This type of division is barely observed in the intact telencephalon (Barbosa et al., 2015; Figure 4E).

## Modulators of Neurogenesis in the Adult Telencephalon

In contrast to embryonic NSCs, which are highly proliferative and differentiate relatively quickly in order to aid the development of the nervous system, the majority of adult NSCs are found in a quiescent state with slow population dynamics (Rothenaigner et al., 2011; Barbosa et al., 2015; Dray et al., 2015). Any alteration of the balance between proliferation and resting stem cells can have drastic consequences and leads to severe neurological disorders. An uncontrolled increase in proliferation of NSCs will lead to premature neurogenesis and consequently to an early depletion of the NSC pool and/or brain tumor formation. In contrast, reduced proliferation will result in a decreased production and supply of new neurons, necessary for brain homeostasis and regeneration (Edelmann et al., 2013). Therefore, understanding the molecular cues controlling adult NSC behavior under regular physiological and pathological conditions is of great importance.

In zebrafish and mouse many signaling pathways, such as Fgf, Shh, Wnt, Insulin, Steroids, BDNF, Notch, and BMP, are involved in adult neurogenesis and control cell quiescence, proliferation and differentiation (Kizil et al., 2012b; Diotel et al., 2013; Urban and Guillemot, 2014; Choe et al., 2015; Horgusluoglu et al., 2017). With respect to recent discoveries, we will first review and compare the role of Notch and BMP signaling pathways in neurogenesis in the germinal zone of the adult telencephalon in zebrafish and mouse. In the second part, we will describe and compare the molecular pathways involved in regenerative neurogenesis in the telencephalon of adult zebrafish and mouse.

## Notch Signaling

**FIGURE 5:**
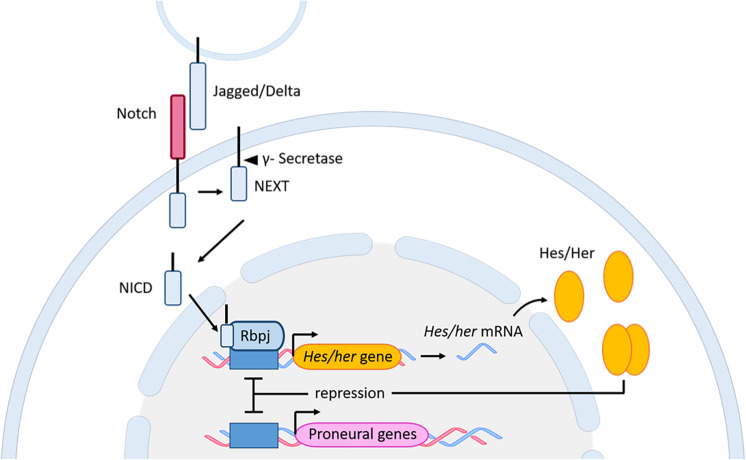
Notch signaling pathway in zebrafish and mouse. Upon interaction between the Notch receptor and its ligands Jagged/Delta, and through several subsequent cleavage steps the NICD is released and translocates to the nucleus. In the nucleus, the NICD, together with Rbpj, activates the expression of the downstream genes *Hes* (mouse) and *her* (zebrafish). The Hes/Her proteins bind as homodimers to the promoter of proneural genes to inhibit neuronal fate. Additionally, the dimer can bind to its own promoter, resulting in a negative auto-regulation. bHLH, basic helix-loop-helix; Her, human epidermal growth factor receptor; Hes, Hairy/enhancer of split; NEXT, Notch extracellular truncation; NICD, Notch intracellular domain; Rbpj, Recombining binding protein suppressor of hairless.

**FIGURE 6:**
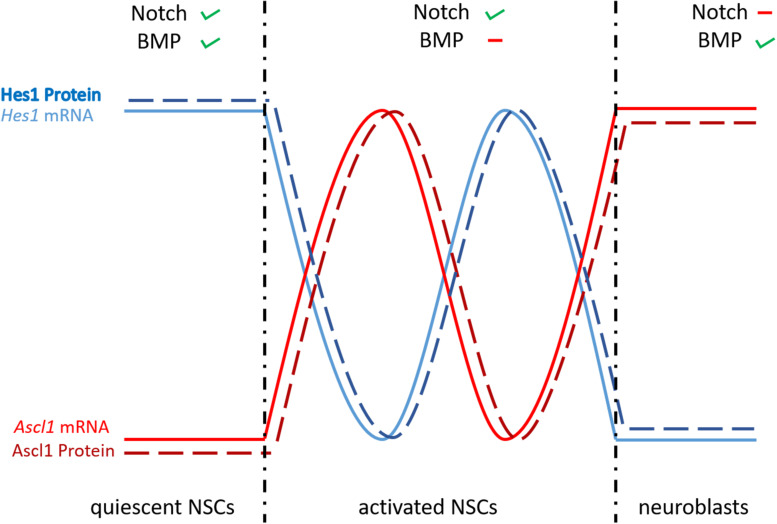
Differential expression of *Hes1* and *Ascl1* controls NSC fate. When BMP and Notch signaling are active, the negative autoregulation of *Hes1* is repressed and its expression is constantly high, leading to quiescent NSCs. The negative autoregulatory effect of Hes1 is high when BMP signaling is not active, causing *Hes1* and *Ascl1* to oscillate. Through this oscillation the NSCs are activated and proliferating. In the case of inactive Notch signaling, the repression of Hes1 on *Ascl1* is abolished and the NSCs develop into neuroblasts. Ascl; achaete-scute like.

The role of the Notch signaling pathway was extensively studied in embryonic neurogenesis where this signaling pathway controls binary cell fate decision by interaction between cellular neighbors (Shin et al., 2007; Kageyama et al., 2008; Cau and Blader, 2009). The Notch signaling pathway is activated by interactions between the Notch receptors and their transmembrane ligands Delta and Jagged (Andersson et al., 2011). Following several cleavage steps, the product, named Notch extracellular truncation (NEXT), is cleaved by a multi-protein complex, called ɣ-Secretase. This cleavage results in the formation of the Notch intracellular domain (NICD) (Artavanis-Tsakonas et al., 1995; van Tetering and Vooijs, 2011), which will translocate to the nucleus to form a complex with the Notch signaling mediator Rbpj (Artavanis-Tsakonas et al., 1999). Finally, this complex activates the expression of downstream genes like members of the hairy/enhancer of split transcription factor family. The most notable member of this family is the mammalian *Hes* genes and its zebrafish homolog *her* (Ohtsuka et al., 1999; Takke and Campos-Ortega, 1999; Takke et al., 1999; Yeo et al., 2007; Figure 5). *Hes*/*her* genes belong to the basic helix-loop-helix (bHLH) family of transcription factors (Davis and Turner, 2001). The basic domain is necessary for binding to their target DNA sequence, while the HLH region forms homo- and heterodimers with other HLH proteins, such as proneural and inhibitor of differentiation (Id) proteins. Hes/Her proteins suppress transcription by binding as dimers to the DNA regulatory sequences of bHLH proneural genes, such as *Achaete-scute like* (*Ascl*), to inhibit their transcriptional expression (Figure 5). BHLH proneural genes are transcriptional activators, which induce the neurogenic fate in the absence of Hes/Her proteins (Bertrand et al., 2002; Fischer and Gessler, 2007; Kageyama et al., 2007a,b; Castro et al., 2011; Imayoshi and Kageyama, 2014b,a). Additionally, during neurogenesis and somitogenesis where the mechanism of Notch signaling in regulating active and quiescent stem cells is very similar (Sueda and Kageyama, 2020), dimers formed by Hes/Her proteins can inhibit their own expression via a negative feedback regulatory loop (Figure 5; Lewis, 2003; Harima et al., 2014). The negative auto-regulation of *Hes/her* genes in combination with the short half-live of Hes/her proteins is responsible for the cyclic expression (oscillation) of Hes/Her transcription factors (Hirata et al., 2002; Shimojo et al., 2008; Oates et al., 2012; Imayoshi et al., 2013). In mouse (not described in zebrafish), the oscillatory behavior of *Hes* genes results in oscillation of *Ascl1* during embryonic neurogenesis (Imayoshi et al., 2013). The level and the type of expression (constant or oscillating) of *Ascl1* determine the behavior of neural progenitor cells. Constant and high expression of *Asc11* is necessary for the commitment of neural progenitor cells to neuronal fate, while its oscillation is indispensable for keeping neural progenitor cells in a proliferative state (Imayoshi et al., 2013; Figure 6).

As already mentioned, bHLH transcription factors interact with the HLH Id protein family through their HLH domain. Because Id proteins lack the basic DNA binding domain (Benezra et al., 1990; Roschger and Cabrele, 2017), heterodimers formed between bHLH and HLH proteins cannot bind or bind with less efficiency to DNA (Massari and Murre, 2000), therefore they behave as dominant negative molecules and inhibit the activity of bHLH transcription factors. For instance, interaction of Id1 with Hes1, one of the key downstream genes of Notch signaling during mouse neurogenesis, inhibits the auto-regulated repression of its own promoter and leads to a persistent expression of *Hes1* (Bai et al., 2007). As a consequence of a high and stable expression of the Hes proteins, the expression of *Ascl1* is constantly repressed. The significance and importance of this mechanism, namely keeping the NSCs in quiescence during adult neurogenesis, will be developed and discussed further in the coming sections.

## Role of Notch Signaling During NSC Homeostasis in the Adult Telencephalon

In zebrafish, a possible role of Notch signaling in adult neurogenesis was first suggested due to the expression of *notch* receptors and *her* genes in neural progenitors of the adult telencephalon. *notch1a/b* and *notch3* and their downstream transcriptional repressor effectors *her4*, *her6* and *her15* are present in RGCs of the adult zebrafish pallium (Chapouton et al., 2010, 2011; Ganz et al., 2010; de Oliveira-Carlos et al., 2013). *notch3* is expressed in both quiescent and activated RGCs, while in contrast, *notch1b* is only expressed in activated proliferating progenitor cells (Alunni et al., 2013; Than-Trong et al., 2018). Genetic approaches, gain- and loss-of-function studies, pharmacological inhibition of Notch and RGC fate tracing suggest that Notch3 signaling promotes both quiescence and the proliferation of RGCs, while Notch1b is necessary to keep the progenitor cells in an undifferentiated proliferative state (Chapouton et al., 2010; Alunni et al., 2013; Than-Trong et al., 2018). Notch3 drives quiescence or proliferation of the progenitor cells via two distinct bHLH downstream mediators, Her4 and Hey1, respectively (Than-Trong et al., 2018). The canonical Notch pathway, mediated by Notch3 and its effector Her4 (the zebrafish ortholog of Hes5 in mouse; see Table 1), leads to the transcriptional repression of proneural genes and consequently to the establishment of quiescent NSCs (Alunni et al., 2013; Kawai et al., 2017). Not much is known why Notch3 in some progenitor cells uses Her, and in others Hey as a mediator of distinct outcomes (quiescence, proliferation, respectively). Additionally, it would be interesting to understand how the expression of Hey is regulated and what are its downstream targets.

The pattern of expression and the function of Notch1 and Notch3 is well conserved between mouse and zebrafish during constitutive adult neurogenesis: (1) Mouse Notch3 is expressed in qNSC and in aNSCs; (2) Knockdown of Notch3 increases the division of NSCs and therefore leads to a reduction of qNSCs; (3) Notch1 is preferentially expressed in aNSCs of the SVZ; and (4) Notch1 deletion in mouse SVZ and SGZ impairs proliferation of NSCs (Ables et al., 2010; Basak et al., 2012; Kawai et al., 2017). However, further investigation is needed to confirm this molecular similarity. In a recent paper, Sueda et al. (2019) showed in mouse that the dual role of Notch signaling in promoting, on the one hand quiescence and on the other hand proliferation of progenitor cells, relies on the concentration of the bHLH transcription factor Hes1 and whether its expression is constant or oscillating (Sueda et al., 2019). They elegantly demonstrated that in qNSCs the expression of Hes1 is kept constantly at a high level (no oscillation), probably because of the inhibition of its own periodical repression by a regulatory feedback loop, involving members of the Id family (Boareto et al., 2017; Harris and Guillemot, 2019; Sueda and Kageyama, 2020). In these cells, the expression of the proneural gene *Asc11* is constantly and fully repressed and therefore cells are maintained in quiescence (Figure 6). In cells, where the Hes auto-regulatory repression is not inhibited, Hes1 and Ascl1 oscillate and the oscillation of Ascl1 promotes activation of NSCs and their proliferation (Figure 6). Finally, in cells without Notch/Hes signaling the expression of *Ascl1* is not periodically repressed and is maintained at a constant level, which causes these cells to differentiate into neuroblasts (Figure 6). Thus, in mouse a quiescent stem cell pool is maintained by the interaction of Id, Hes, and Ascl1. It is possible that the same mechanisms exist in zebrafish, since key players are also expressed in the ventricular zone of the telencephalon in adult zebrafish (Notch, Id, Ascl1, and Her). This hypothesis is supported by the work of Rodriguez Viales et al. (2015) which showed that Id1 keeps NSCs in quiescence during constitutive and regenerative neurogenesis (Rodriguez Viales et al., 2015). In agreement, recombinant zebrafish Id1 preferentially interacts with Her4, 6, and 9 proteins. Notch involvement was shown already in quiescent but also proliferating progenitor cells via a Notch3/Her and Notch3/Hey-axis, respectively (Than-Trong et al., 2018). Still the question remains, whether an Id-Her-Ascl pathway would work in parallel or in addition to the Notch/Her and Notch/Hey pathways in zebrafish. To decipher the role of Notch in controlling the behavior of NSCs further investigation is needed, focusing on a possible oscillatory expression of one or several of the zebrafish Her and Ascl proteins.

## Bone Morphogenetic Protein Signaling Pathway

**FIGURE 7:**
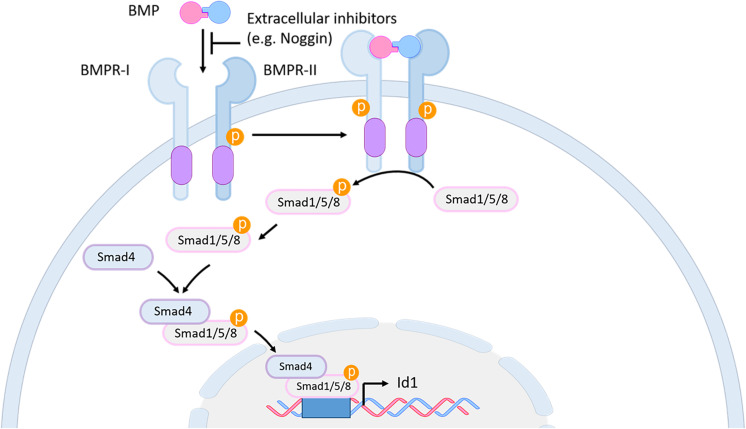
BMP signaling pathway in mouse and zebrafish. BMP proteins bind as dimers to a transmembrane receptor complex, formed by BMPR-I and BMPR-II. Through this binding, which can be blocked by extracellular inhibitors like Noggin, a phosphorylation cascade is initiated leading to the phosphorylation of the Smad1/5/8 proteins. The activated Smad1/5/8 forms a complex with Smad4 which interacts in the nucleus with specific co-factors to bind to the regulatory sequences of downstream genes, such as *id1*. BMP, bone morphogenetic protein; BMPR, BMP receptor; id1, inhibitor of DNA binding 1.

Bone morphogenetic proteins (BMPs) are members of the transforming growth factor β (TGF-β) family (Miyazono et al., 2010). BMP family members are secreted signaling molecules that act in a dose-dependent fashion to regulate many developmental and adult homeostatic processes (Wang et al., 2014; Bier and De Robertis, 2015). Secreted BMP proteins form homo- or heterodimers, which are activated after proteolytic cleavage by a serine protease (Cui et al., 1998). A balance between active BMPs and their secreted antagonist molecules, such as Noggin, Chordin, and Follistatin in part establishes the local concentration and availability of BMP proteins. Mature BMP dimers bind to a heterotetrameric complex of transmembrane type I and type II serine/threonine receptors. This binding triggers a cascade of protein phosphorylations, which eventually lead to the phosphorylation of BMP-subfamily-specific Smad proteins (Smad1/5/8) and their association forming a complex with the Co-Smad Smad4, which is a common downstream mediator of all TGFß subfamily members. The resulting heteromeric Smad complex translocates to the nucleus, where it interacts with cell/tissue specific cofactors and binds to the regulatory sequences of downstream target genes to regulate their transcriptional expression (Shi and Massague, 2003; Figure 7).

Inhibitor of differentiation (*id*) genes are among the best characterized direct BMP target genes (Hollnagel et al., 1999). Most *id* genes contain an evolutionary conserved BMP-responsive element (BRE) including characteristic Smad and Smad-cofactor binding motifs (Korchynskyi and ten Dijke, 2002; Lopez-Rovira et al., 2002; Karaulanov et al., 2004; Nakahiro et al., 2010; Javier et al., 2012; Zhang et al., 2020).

In zebrafish, the Id HLH protein family of transcriptional regulators contains five members, whereas in mouse there are only four (Ling et al., 2014; Diotel et al., 2015a). The Id HLH proteins behave as repressor molecules by forming hetero-dimeric protein complexes with bHLH transcription factors which, as a consequence, cannot or only weakly bind to their targeted DNA binding sites (Benezra et al., 1990; Ishii et al., 2012). Id proteins have overlapping and distinct functions during development and body homeostasis. They control the cellular state, fate and migration of different cell types (Ling et al., 2014).

## The BMP Signaling Pathway and Its Role in Adult Neurogenesis

The involvement of BMP signaling in neurogenesis of adult zebrafish was mainly demonstrated through the investigation of the regulation and function of its downstream target gene *id1*. Zebrafish *id* genes are expressed in the adult zebrafish brain in an overlapping manner (Diotel et al., 2015a). However, only *id1* expression is restricted to the ventricular zone of the telencephalon where its expression is mainly found in quiescent RGCs. Over-expression of *id1* leads to quiescence of NSCs, while morpholino-mediated *id1* knockdown increases the number of proliferating RGCs and favors neurogenesis. These gain- and loss-of-function studies demonstrate the importance of Id1 in maintaining the balance between dividing and resting NSCs by promoting RGC quiescence (Rodriguez Viales et al., 2015). Furthermore, an evolutionarily conserved cis-regulatory module (CRM) of *id1* was identified which mediates specific expression of *id1* in RGCs. Investigation of this CRM by systematic deletion mapping, mutations of the binding sites for BMP pathway members like Smad transcription factors, as well as pharmacological inhibition of Smad phosphorylation and transcriptome analysis, suggest a crucial role for BMP signaling in controlling *id1* expression in RGCs and their quiescence in the zebrafish adult brain (Zhang et al., 2020).

A very similar function of the *id1* gene in controlling adult NSC quiescence is described in the mouse SVZ, where this gene is highly expressed in the quiescent B1 cells (adult NSCs) to control their self-renewal capacity (Nam and Benezra, 2009). The role of BMP signaling in mouse, in terms of NSCs and neurogenesis is complex and subject to discussion. In the mouse adult SGZ, the antagonistic interplay between BMP and its inhibitor Noggin, as well as the differential expression of the BMP receptor Ia were shown to be necessary for the maintenance of the quiescent state of the adult NSCs (Lim et al., 2000; Mira et al., 2010; Bond et al., 2014). Although, in mouse SVZ, *in vitro* and *in vivo* data have shown that BMP signaling inhibits neurogenesis (Lim et al., 2000; Choe et al., 2015), another work in which Smad4 was conditionally inactivated contradicted these findings and argued for a role of BMP signaling in neurogenesis rather than keeping the stem cells quiescent (Colak et al., 2008). To reconciliate these contradictory findings, Choe et al. (2015) proposed that in the SVZ, BMPs work very early when the fate of the adult NSCs is being established. According to this model, there is a very subtle difference in BMP concentration that might decide which cell fate the stem cell progenies will adopt. In the SVz, a local BMP gradient is established by the BMP antagonist Noggin, released by the ependymal cells of the telencephalic ventricle. Thereby, NSCs close to the ventricular zone will be exposed to a lower concentration of BMP while cells further away will be in contact with a higher concentration of BMP. As a consequence, cells which receive high BMP signaling will express a high level of the BMP target gene *id1* and will transform into quiescent NSCs (type B). In contrast, when NSCs are exposed to a lower level of BMP signaling, they will also express lower levels of *id1* and become activated to later either differentiate into astrocytes, neuroblasts or oligodendrocytes (Choe et al., 2015). In this second phase of cell differentiation the presence or absence of BMP is again crucial to determine the final cell fate (Choe et al., 2015).

**FIGURE 8:**
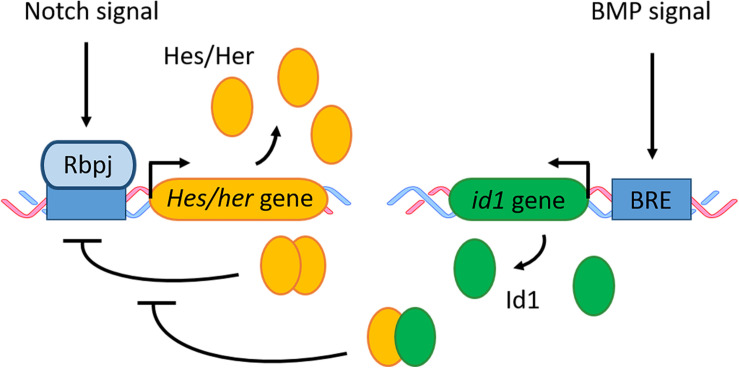
BMP and Notch signaling in quiescent RGCs/NSCs. Notch signaling activates gene transcription of *Hes* in mouse and its zebrafish ortholog *her.* The Hes/Her proteins form a homodimer which can bind to its own promoter and therefore block its own transcription in a negative feedback loop. BMP signaling activates the transcription of its downstream effector *id1*. The Id1 protein forms a complex with Hes/Her which inhibits the negative regulatory feedback loop of Hes/Her. BRE, BMP responsive element.

The mechanism by which *id1* promotes adult neural stem cell quiescence in zebrafish and mouse can be explained through the interaction of *id1* with members of the Hes/Her protein family (Figure 8). Indeed, GST pull-down experiments showed that Id1 proteins interact with Her4.1, Her4.5, Her6, and Her9 proteins *in vitro* (Rodriguez Viales et al., 2015). Id1 can form a dominant negative complex with Hes and Her, which behaves like an antagonist of the Hes/Her dimer complexes and prevents binding of the dimer to its target DNA. For instance, interaction of Id1 with Hes in mouse interferes with the Hes mediated negative auto-regulation of its own promoter, leading to a high and constant expression of Hes in NSCs and therefore to quiescence of the NSCs (Bai et al., 2007; Sueda and Kageyama, 2020; Figures 6, 8), suggesting an interaction of the BMP and Notch pathways in the mouse telencephalon. Such a functional synergy between Id1 and Her (Hes) proteins has not been demonstrated in the zebrafish yet. However, BMP and Notch signaling effectors and targets are expressed in a similar pattern in adult NSCs in both species. Furthermore, the observed phenotypes in gain-of-function and loss-of-function experiments of the BMP and Notch signaling mediators (Id1, Hes, and Her) are very similar in both species (Rodriguez Viales et al., 2015; Boareto et al., 2017; Sueda and Kageyama, 2020). Therefore, it is tempting to speculate that in zebrafish, like in mouse, BMP and Notch signaling are crucial in controlling adult neural stem cell behavior. Many aspects of the molecular mechanisms controlling adult neurogenesis appear to be conserved between mouse and zebrafish (Table 1).

## Molecular Mechanisms of Adult Zebrafish Telencephalon Regeneration

Several traumatic brain injury and neurodegenerative disease models have been developed in adult zebrafish. Among them, telencephalic stab wound injuries (Kroehne et al., 2011; März et al., 2011; Baumgart et al., 2012; Kishimoto et al., 2012; Schmidt et al., 2014) and Alzheimer’s disease models (Bhattarai et al., 2016, 2020; Cosacak et al., 2019) are certainly the best understood ones at the cellular and molecular level.

In the stab injury paradigm, the adult zebrafish telencephalon is damaged by pushing a needle either through the skull into the medial region of the telencephalon (März et al., 2011; Kishimoto et al., 2012; Schmidt et al., 2014) or through the nostrils to reach the parenchyme of the telencephalon (Kroehne et al., 2011; Baumgart et al., 2012). With these methods only one telencephalic hemisphere is injured while the contralateral hemisphere is kept intact and can serve as a control. In the past the stab wound injury, combined with transcriptomic techniques and RNA *in situ* hybridization, identified several hundred genes with a potential role in zebrafish adult neurogenesis and regeneration (Kizil et al., 2012b; Rodriguez Viales et al., 2015).

Upon telencephalic injury the expression of *gata3*, a zinc finger transcription factor is increased in RGCs and promotes their proliferation and differentiation into neurons (Kizil et al., 2012b). Consistently, inhibition of Gata3 activity via morpholino injection into the brain ventricle leads to a decrease in injury-induced proliferation of RGCs and to blunted neurogenesis (Kizil et al., 2012b). This injury-mediated induction of Gata3 and subsequent neurogenesis was shown to be dependent on inflammation. In agreement with this finding, cerebroventricular microinjection of Zymosan A, an inflammatory compound from Saccharomyces cerevisiae, results in (1) an increased expression of pro-inflammatory cytokines tnfa (tumor necrosis factor-α), il1ß (interleukin 1ß) and il8 (interleukin 8) in the injured hemisphere; (2) an increased proliferation of S100ß-positive RGCs; (3) induction of *gata3* expression at the ventricular zone and (4) a higher number of newborn HuC/D-positive neurons generated 21 days post injection (dpi) (Kyritsis et al., 2012). Additionally, it was shown that in response to inflammation the leukotriene C4 (LTC4) and its receptor CysLT1 were able to trigger the activation of Gata3 in the injured telencephalon (Kyritsis et al., 2012). These findings show that brain inflammation, including the leukotriene signaling pathway is sufficient to initiate regenerative mechanisms for reactive neurogenesis in the zebrafish telencephalon (Kyritsis et al., 2012).

The expression of the transcriptional regulator *id1* is also up-regulated in NSCs after injury (Rodriguez Viales et al., 2015). However, in contrast to *gata3*, the induction of *id1* expression does not depend on inflammatory signals (Rodriguez Viales et al., 2015) but on the BMP signaling pathway, which is necessary to drive quiescence of NSCs (Zhang et al., 2020). Functional analysis of *id1* under regenerative conditions, suggested that Id1 counteracts injury-induced neurogenesis by promoting stem cell quiescence, and thereby maintaining the neural stem cell pool by avoiding its exhaustion (Rodriguez Viales et al., 2015).

After traumatic lesion, the expression of Notch receptors and some of their downstream mediators are increased in NSCs (Kishimoto et al., 2012; Rodriguez Viales et al., 2015). The regulation of the Notch pathway, as well as the exact role of Notch signaling during injury-induced neurogenesis are not well understood and are subject of discussion. Because *notch1* expression is increased in proliferating progenitors after telencephalic lesion, and inhibition of the Notch pathway in injured animals leads to a reduction in the number of neural progenitor cells (NPCs), Kishimoto et al. (2012) suggested that Notch1 signaling is crucial for injury-induced proliferation and neurogenesis in the zebrafish telencephalon. However, no direct evidence for a particular function of Notch signaling in neurogenesis was provided. From what is known about the role of Notch1 during constitutive neurogenesis [Notch 1 controls progenitor proliferation in constitutive neurogenesis (Alunni et al., 2013)], it is possible that the reduction in the number of NPCs observed by Kishimoto et al. (2012) is simply due to a reduction in proliferation upon Notch inhibition. Still, it cannot be excluded that BMP and Notch signaling pathways are induced simultaneously and interact with each other to promote progenitor quiescence using the same mechanism as in constitutive neurogenesis (Sueda et al., 2019).

In summary, a lesion of the zebrafish telencephalon leads to an acute inflammatory response and injury-induced neuroinflammation in zebrafish acts as a positive neurogenic signal, which activates Gata3-mediated neurogenesis. The activation of the BMP/Id1 signaling pathway is delayed compared to *gata3*. In this manner, BMP/Id1 counteracts the neurogenic program initiated by Gata3, through promoting neural stem cell quiescence, therefore avoiding excessive production of new neurons, which subsequently would deplete the stem cell pool. In the zebrafish telencephalon, regeneration is mediated by the activation and combination of specific injury-dependent (inflammatory) pathways and constitutive neurogenic pathways (BMP/Notch). Thus, the regenerative program occurring in the telencephalon of adult zebrafish after injury involves several signaling and molecular pathways, acting together in a complex way. These signals and regenerative programs would allow the genesis of new neurons, their migration, differentiation and integration within the damaged telencephalic area.

## Responses to Traumatic Brain Injury in Mouse

Although, the early phases in posttraumatic mouse and zebrafish models of injury-induced signaling pathways are very similar, their functions seem to be aimed at totally opposite directions. In mouse, the role of inflammation in NSC activity is subject to debate. It can have both a positive and a negative effect on these cells which is context and time dependent (Kizil et al., 2015). It can have an inhibitory effect on neurogenesis and brain regeneration through the inhibition of NSC proliferation (Ekdahl et al., 2003; Monje et al., 2003; Iosif et al., 2006), while simultaneously activating the proliferation of quiescent glial cell populations such as astrocytes and microglia (Buffo et al., 2010). However, the pro-inflammatory cytokines and chemokines released during brain injury in mammals can also favor neurogenesis causing different effects on NSC proliferation, neuronal differentiation, migration and survival (Carpentier and Palmer, 2009; Whitney et al., 2009; Covacu and Brundin, 2017). An increase in the number of glial cells impacts glia-neuron and glia-glia interactions and consequently leads to axonal degeneration and neuronal death (Bal-Price and Brown, 2001). Astrocytes are critical actors in the post-traumatic response and are involved in different response mechanisms, among them the formation of a glial scar. Furthermore, they secrete different cytokines or proteoglycans, which promote neurotoxicity and inhibit axon regeneration, respectively (Yiu and He, 2006; Rao et al., 2012; Anderson et al., 2016; Burda et al., 2016). However, the formation of the glial scar can also be beneficial as its inhibition worsens CNS damage (Anderson et al., 2016). In addition, neuroinflammation in mammals is a long- lasting process and often the acute phase is followed by a chronic phase which can foster many neurodegenerative diseases, such as Parkinson’s and Alzheimer’s (Amor et al., 2010; Glushakova et al., 2014; Kinney et al., 2018). The role of inflammation is consequently complex in the CNS of mammals and is spatio-temporally dependent on the regenerative processes and the types of damage (ischemia, trauma, and neurodegenerative diseases, etc.).

Interestingly, BMP and Notch signaling are also induced after injury in mouse, but in contrast to zebrafish they induce glial cell fate (Bohrer et al., 2015; Pous et al., 2020). Accordingly, in the injured mouse brain, the progeny of SVZ NSCs will not migrate to the OB to differentiate into neurons, but instead these cells are recruited to the injury site where they differentiate into glia and participate in the formation of a glial scar, which will subsequently repress neural regeneration (Goings et al., 2004; Wang et al., 2018). It was shown in mouse that upon traumatic brain injury and consequent vascular rupture, the coagulation factor Fibrinogen is released into the SVZ where it activates BMP signaling and increases the expression of its direct downstream mediator Id3 (Pous et al., 2020). Injury-induced up-regulation of Id3 by BMP2 in the SVZ promotes astrogenesis over neurogenesis by positively regulating the expression of astrocyte specific genes such as GFAP, GLAST, and Aldh1l1. The transcriptional regulator Id3 forms heterodimers with the bHLH transcription factor E47 and thereby inhibits the binding of E47 to the regulatory sequence of astrocyte specific genes like GFAP. Through this mechanism, E47 mediated repression of astrocyte specific genes is abolished and the NSCs preferentially differentiate into astrocytes (Bohrer et al., 2015).

Likewise, injury-induced *Notch* expression promotes astrogenesis in the adult mouse SVZ (Tatsumi et al., 2010; Benner et al., 2013). Here, activation of the Notch pathway in the injured brain was shown to be up-regulated by thrombospondin 4 (Thbs4), a secreted homopentameric glycoprotein produced by a sub-population of SVZ astrocytes that binds to the Notch1 receptor (Adams and Lawler, 2011; Benner et al., 2013).

In summary, although traumatic brain injury in the mouse model activates a combination of injury-induced inflammation molecules and signaling pathways, implicated in constitutive neurogenesis, such as BMP and Notch which is similar to adult zebrafish, their activation in mammals favors astrogenesis over neurogenesis. Understanding why in different vertebrate species, molecular pathways that are initiated after brain injury have different functions and opposite outputs on neurogenesis and regeneration, will be a key step to selectively activate the necessary regenerative program at the right time to promote regeneration without scar formation.

## Neural Regeneration in Alzheimer’s Disease Model in Zebrafish

Alzheimer’s disease is a neurodegenerative disorder in which neurons are progressively dismounted and die. Neural death and synaptic impairment are partially due to deposition and accumulation of Amyloid beta (Aβ) peptides around and in neurons (Hardy and Higgins, 1992; Gouras et al., 2000; Bayer and Wirths, 2010). Furthermore, neural death is generally accompanied by a reduction in neurogenesis, which progressively results in impaired memory, thinking, and confusion (Moreno-Jimenez et al., 2019).

To investigate the molecular mechanisms involved in zebrafish brain regeneration and NSC plasticity after loss of neurons, Kizil and colleagues established an induced Alzheimer’s disease model by injection of cell- penetrating Amyloid-β42 (Aβ42) into the ventricle of the adult zebrafish telencephalon (Bhattarai et al., 2016). Interestingly, in its early phase Aβ42 injection and subsequent aggregation in neurons led to a neurodegenerative phenotype, similar to what is observed in human Alzheimer’s patients. The injected fish showed Aβ42 deposition and accumulation, cell death, inflammation, synaptic degeneration and learning impairment. In a later phase, Aβ42 injected fish exhibited increased NSC proliferation and neurogenesis. Transcriptome analysis of the injected fish revealed an up-regulation of the cytokine interleukin-4 (IL4) and an increase in the phosphorylation of its downstream effector- STAT6, suggesting that in the zebrafish model of induced Alzheimer’s pathology, the IL4/STAT6 regeneration program is necessary for RGC proliferation and their subsequent differentiation into functional neurons (Bhattarai et al., 2016). Further investigation using a single cell sequencing approach demonstrated that IL4/STAT6-mediated neuroregeneration involves inhibition of the anti-proliferative effect by serotonin. The latter is an inhibitor of Brain-derived neurotrophic factor (BDNF), which promotes NSC proliferation and differentiation (Bhattarai et al., 2020).

These findings, together with earlier results regarding the role of LTC4/Gata3 in brain regeneration after traumatic injury, indicate that the zebrafish responds to different types of brain damage with different neuroregenerative mechanisms.

However, none of these regenerative programs in zebrafish have been identified in mammals, so far. No up-regulation of Gata3 after injury could be observed in the rodent SVZ (Yoshiya et al., 2003; Chang et al., 2016) or in primary human astrocytes in 3D culture (Celikkaya et al., 2019). Accordingly, in the DG of the mouse Alzheimer’s disease model, the expression of the interleukin-4 receptor (IL4R) could not be induced in astrocytes (Mashkaryan et al., 2020).

It thus remains to be seen whether these are genuine differences that could account for the limitations of the mammalian brain to repair neurodegenerative lesions of the CNS effectively.

Interestingly, when human astroglia cells expressing IL4R in culture were activated by the ligand IL4, they showed active Type 2 IL4 signaling and enhanced proliferation, neurogenesis and neuronal network formation under Alzheimer’s disease conditions, which is reminiscent of the zebrafish Alzheimer’s model (Papadimitriou et al., 2018). However, other data show that neurogenesis during regeneration in mammals cannot simply be induced by exogenous activation of zebrafish regenerative programs. For example, over-expression of *Gata3*, the immediate early player in the traumatic injury response in zebrafish, increases the neurogenic potential of primary human astrocytes in 3D culture without leading to neurogenesis (Celikkaya et al., 2019).

The difference between zebrafish and mammals, with respect to the regenerative ability might be explained by (1) the incapacity of the mammalian nervous system to activate programs which lead to the coordinated proliferation of NSCs in response to traumatic and neurodegenerative damage of the CNS, (2) the difference in transcriptional and signaling networks in NSCs between zebrafish and mammals, (3) the difference in the nature of prospective NSCs in these distinct models.

## Conclusion and Perspectives

Recent progress demonstrated many similarities at the cellular and molecular level between mice and zebrafish regarding adult neurogenesis. Both species employ the same signaling systems (Notch, BMP) and related downstream mediators (Id1, Hes/Her) to control the proliferation and differentiation of adult NSCs (Table 1). The activity of the individual pathways but possibly also their synergy appears to determine quiescence, proliferation and differentiation of RGCs in both mammals and zebrafish. Strong activity, coupled with direct interaction between BMP and Notch downstream mediators, Id and Hes/Her proteins, promote quiescence. Reduction in activity strength or absence of one or both signals leads to NSC proliferation or neural differentiation.

A major difference appears to be the scale at which adult neurogenesis occurs in the two organisms. In the adult zebrafish brain, almost all sub-domains of the brain are competent to generate new neurons, while the neurogenic potential is largely restricted to two domains in the mouse forebrain (SVZ and SGZ). Furthermore, in zebrafish, the higher proliferative capacity is correlated with the presence of RGCs during adult stages. These cells span, with their long processes, the entire distance through the telencephalic parenchyme from the ventricular to the pial surface, similar to the RGCs present in the nascent mouse telencephalon in prenatal stages. Adult zebrafish RGCs, as well as the NSCs in the adult mouse brain are related to astroglia and express highly similar astroglial markers. In the mouse, the prenatal RGCs are transformed largely into astrocytes, a cell type that is not present in the zebrafish brain. Taken together, it appears that the adult zebrafish telencephalon with its less complex structure has retained embryonic structural and functional features permitting neuronal production at large scale in the mature brain. In mammals, RGCs have evolved into novel cellular components (astrocytes) having thereby lost their ability to generate neurons. The NSCs in the remaining stem cell niches of the murine telencephalon retained the astroglial characteristics of the RGCs but have lost their parenchyme-spanning features, possibly as an adaption to the structurally much more complex brain of mammals.

Another major difference between mammals and zebrafish is the response to brain tissue damage and concomitantly the ability to repair damaged nervous tissue. Although in zebrafish, NSCs rapidly increase their production of new neurons in response to both traumatic and neurodegenerative nervous tissue damage, the predominantly newly produced cell type in the damaged mammalian brain is astrocytes. Furthermore, while in zebrafish newborn neurons are able to integrate efficiently into the damaged tissue without scaring, in mammals scar formation prevents functional restoration of the nervous tissue. Thus, the overproduction of astrocytes in response to injury and the resulting gliosis with permanent scaring are crucial cellular differences between zebrafish and mammals.

Interestingly, distinct pathways trigger tissue repair in response to traumatic injury and neurodegeneration in zebrafish, suggesting parallel pathways to deal with different types of tissue damage. Traumatic brain injury relies on the LTC4/Gata3 program, while the induced neurodegenerative Alzheimer’s disease depends on the IL4/STAT6 route. These pathways appear to serve as triggers for increased neurogenesis which utilizes mechanisms from constitutive neurogenesis (BMP and Notch) to manage and maintain stem cell pools. In contrast to zebrafish, the inflammatory signals in mammals lead to activation of astrocytes and ultimately gliosis. Furthermore, the IL4/STAT6 pathway has so far not been successfully used *in vivo* in mammalian systems to trigger tissue regeneration (Mashkaryan et al., 2020). For therapies, it is thus essential to find ways to reprogram astrocytes to behave like their ancestral RGCs, in order to produce mainly neurons that integrate into the functional neural circuit.

In conclusion, zebrafish and mouse constitutive and regenerative neurogenesis display some common features considering the type and the nature of NSCs as well as the signaling pathways controlling their activity. A better understanding of the molecular cues and mechanisms sustaining the strong neurogenic capability of NSCs in zebrafish would help to develop new therapies to combat the debilitating consequences of brain damage.

## Author Contributions

SR and ND conceived the idea of this review. SR, ND, US and LL wrote the manuscript together. All authors contributed to the article and approved the submitted version.

## Conflict of Interest

The authors declare that the research was conducted in the absence of any commercial or financial relationships that could be construed as a potential conflict of interest.
